# Application of Natural Plant Fibers in Cement-Based Composites and the Influence on Mechanical Properties and Mass Transport

**DOI:** 10.3390/ma12213498

**Published:** 2019-10-25

**Authors:** Kaiyue Zhao, Shanbin Xue, Peng Zhang, Yupeng Tian, Peibing Li

**Affiliations:** Center for Durability & Sustainability Studies of Shandong Province, Qingdao University of Technology, Qingdao 266033, China; applezkyzhao@163.com (K.Z.); shanbin_xue@163.com (S.X.); 17853245870@163.com (Y.T.);

**Keywords:** pineapple leaf fiber, ramie fiber, mechanical properties, flexural toughness, capillary absorption, chloride penetration

## Abstract

Recently, there is ongoing interest in the use of natural plant fibers as alternatives for conventional reinforcements in cementitious composites. The use of natural plant fibers makes engineering work more sustainable, since they are renewable, biodegradable, energy-efficient, and non-toxic raw materials. In this contribution, a comprehensive experimental program was undertaken to determine the influence of pineapple leaf fiber and ramie fiber on the mechanical properties and mass transport of cement-based composites. The compressive strength, tensile strength, modulus of elasticity, modulus of rupture, fracture energy, flexural toughness, coefficient of capillary water absorption, and chloride diffusion were measured. Natural plant fiber-reinforced cement-based composites (NPFRCCs) containing pineapple leaf fiber and ramie fiber, as compared to the plain control, exhibited a slight reduction in compressive strength and a considerable improvement in tensile strength, modulus of elasticity, modulus of rupture, and flexural toughness; the enhancement was remarkable with a higher fiber content. The coefficient of capillary absorption and chloride diffusion of NPFRCCs were significantly larger than the plain control, and the difference was evident with the increase in fiber content. The present study suggests that the specimen with 2% pineapple leaf fiber content can be used in normal environments due to its superior mechanical properties. However, one should be careful when using the material in marine environments.

## 1. Introduction

Sustainability is defined by the World Commission on Environment and Development as the ability to meet current needs without compromising the ability of future generations to meet their own needs [1]. One major problem facing mankind is the growing world population and the related pressure on construction environment [2]. Fibers, mainly including metal fibers, synthetic fibers, and natural fibers, are widely used today for building reinforcement of cementitious composites. The existing literature is replete with studies showing that the incorporation of metal fibers or synthetic fibers can significantly enhance tensile strength [3,4,5], toughness [6,7], ductility [8], crack resistance [9], and impact resistance [10] of cement composites.

However, the production process of the aforementioned fibers consumes a huge amount of fossil energy and the contradiction between performance and price of these fibers cannot be effectively solved. Natural plant fibers represent a commendable solution to the above issues with their abundant supply and the characteristics of environmental protection, energy saving, and degradable regeneration [11,12,13,14,15].

Many attempts were made to develop natural plant fibers in cement-based materials. Fiber treatments are used to mitigate the high water absorption of cellulose fibers. Fiber hornification is one of the commonly used fiber treatment methods, which implements wetting and drying cycles, resulting a higher dimensional stability and, through which, the fiber–matrix interface [16], mechanical performance, and durability [17] of cementitious materials are improved. In the uniaxial tensile test, the curauá fiber outperformed jute, coir, sisal, and piassava fibers in tensile strength and Young’s modulus [18]. The physical and mechanical properties [19,20], fracture and flexural properties [21], and fatigue behavior [22] of natural fiber-reinforced composites were comprehensively studied. Promising conclusions were drawn stating that the incorporation of natural plant fibers is beneficial in the physical, mechanical, and flexural behaviors of cement-based materials. Meanwhile, in previous studies [23,24] on sisal fibers in cement-based composites, a multiple cracking process with a strain-hardening behavior in both tension and bending was observed. The strain-hardening phenomenon was also observed in alkali-treated natural curauá fiber cementitious composites [25]. Meantime, Bartosz found a strain-softening behavior of alkali-treated natural curauá fiber exposed to weathering in his other study [26] and attributed it to deterioration of the fiber–matrix bond. The conclusions of these studies suggested the replacement of synthetic fibers and metal fibers with natural plant fibers.

Pineapple is a non-climacteric crop; it is the third most widely cultivated plant, and its huge output causes a lot of agricultural waste to be handled [27,28]. Furthermore, among various natural fibers extracted from plant leaves, pineapple leaf fiber (PALF) has the highest cellulose content and a low microfibrillar angle, which are dominant factors attributing to the improvement of its tensile properties [29]. Meanwhile, ramie fiber (RF) gained sufficient attention as it is easily available and possesses considerable tenacity and tensile strength, which contributes tremendously to its potential use in various engineering applications [30].

The industrial production of RF and PALF by chemical degumming results in problems such as environmental pollution and inferior fiber quality, making them unsustainable and hindering the development of relevant industries [31]. Several approaches were undertaken to address the application of PALF and RF. Using an alkali and silane treatment, impurities were removed from PALF, and it displayed better tensile strength [32]. Through the introduction of *Bacillus* sp. HG-28 in ramie fibers, the residual gum content, and the consumption of chemicals and water were significantly reduced [31]. Moreover, the tensile strength, tensile modulus, elongation at break, and bending strength were significantly increased with the increase in PALF content compared to a polypropylene matrix [33]. The flexural and impact properties of polyester and epoxy composites were also found to notably improve with the increase in PALF content [34].

Overall, natural plant fiber-reinforced cement-based composites (NPFRCCs) are superior in toughness, ductility, and impact resistance properties. However, relatively less attention was paid to their durability, especially studies related to water and chloride penetration in the PALF- and RF-reinforced cement-based materials. The limited research on this relatively new composite material indicated the formation of a potentially tougher and more ductile system [35,36,37,38].

Therefore, this paper aimed to study the mechanical and mass transport characteristics of cementitious composites reinforced with PALF and RF. Natural plant fibers were firstly soaked in an NaOH and Ca(OH)_2_ solution with a pH value of 12 for seven days, 30 days, and 60 days; they were then dried in an oven at 100 °C, and the tensile strength and fiber morphology were analyzed. Subsequently, the uniaxial tensile test, three-point and four-point bending test, capillary absorption test, and chloride penetration test were carried out. The compressive strength, tensile strength, modulus of elasticity, modulus of rupture, fracture energy, flexural toughness coefficient of capillary water absorption, and chloride diffusion were then evaluated for the natural fiber-reinforced samples.

## 2. Materials and Methods

### 2.1. Materials and Mix Proportions

The mix proportions of cement-based composites are listed in Table 1. Ordinary Portland cement (OPC) 42.5 produced by Qingdao Shanshui Co. Ltd. (Qingdao, China) was prepared with a water/binder ratio of 0.33. Class I fly ash produced by Nanjing Electric Heating Factory was used as one of the main binders, and the particle size of the silica sand produced locally in Qingdao was less than 0.3 mm. The superplasticizer produced by Jiangsu Sobote New Materials Co. Ltd. (Nanjing, China) had a higher water reduction rate of 15% to 30%. The chemical compositions of OPC and fly ash are summarized in Table 2.

**Figure 1:**
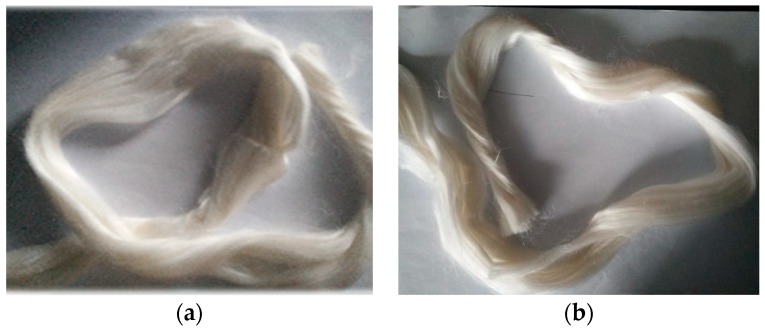
The apparent morphology of pineapple leaf fiber (PALF) (**a**) and ramie fiber (RF) (**b**).

**Figure 2:**
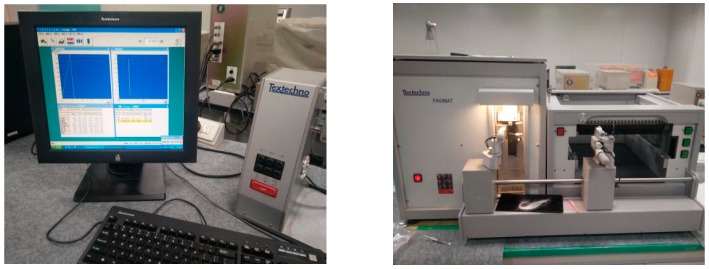
Favimat-Airobot automated monofilament fiber tester.

Natural plant fibers, including pineapple leaf fiber and ramie fiber, used in this project were obtained as fine, dry, long fibers from Qimun Tianan Textile & Garment Co. Ltd. (Huangshan, China) in Anhui Province, as shown in Figure 1. The fine, dry, long fibers were produced through a degumming process (acid leaching, alkali boiling, re-acid leaching, re-alkali boiling, and refining) from the original fiber. The original fiber was obtained using a 6BZ-400 decorticator to mechanically strip the pineapple leaves and ramie leaves. The long fibers were cut into short fibers with a length of 15 mm before being incorporated into the fresh mixture. The properties of the natural plant fibers obtained from the Favimat-Airobot automated monofilament fiber tester are summarized in Table 3, and the apparatus of the tester is shown in Figure 2.

### 2.2. Preparations for Test Specimens

**Figure 3:**
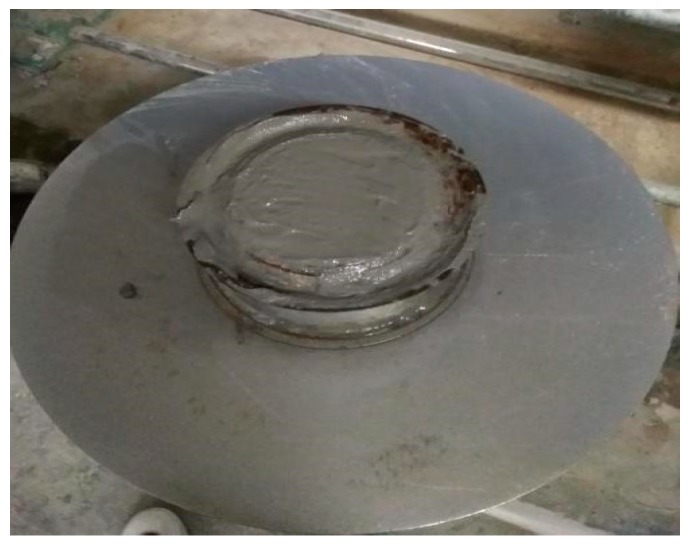
Slump test of P1.

The mixing procedure for all specimens was designed to disperse PALF and RF uniformly, which largely depended on the fiber type and stirring process. The solid dry raw materials were mixed for 2 min. Water and polycarboxylic acid superplasticizer were then added and stirred for 2 min to acquire better fluidity. Thereafter, fibers were gradually added and mixed in the cement mortar mixer produced by the Wuxi Jianding construction instrument factory (Wuxi, China) at a low speed of 140 rpm for 4 min and a high speed of 285 rpm for another 4 min until a uniform fiber dispersion was observed. Figure 3 shows the slump test of P1, and a uniform dispersion of pineapple leaf fibers was observed during the test. After mixing, the fresh mixture was placed into molds. Specimens were compacted with a vibrating table to ensure proper consolidation and to minimize the amount of entrapped air. All specimens were cured in a standard curing room for a specific time.

### 2.3. Uniaxial Tensile Test

**Figure 4:**
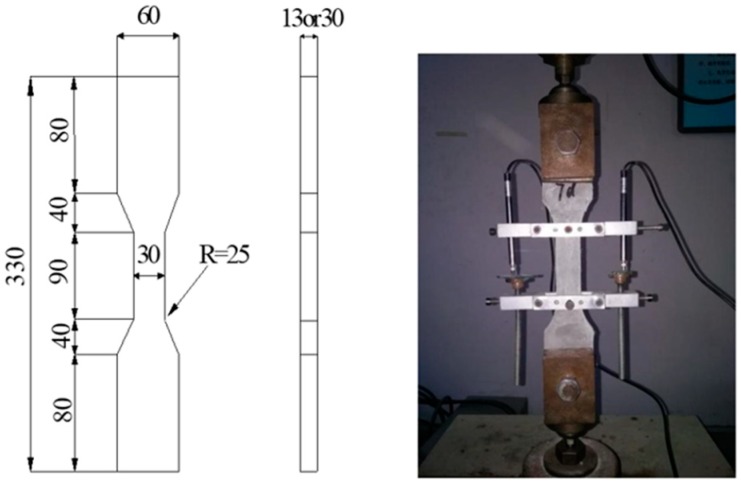
Dog-bone-shaped specimen for uniaxial tensile test (all dimensions in mm).

Full-range stress–strain curves were obtained under a uniaxial tensile test with a dog-bone-shaped specimen, as shown in Figure 4. The test was carried out on an AG-TS testing machine (Shimadzu, Kyoto, Japan) with 250-kN load-cell capacity under a constant loading rate of 0.1 mm/min. Two linear variable displacement transducers (LDVTs) affixed to a testing frame were used to measure the deformation of specimens. Two ends of the specimen were fixed in the testing machine, with two chucks engraved with horizontal stripes to enhance friction. The tensile strain was obtained through the average reading of the two LDVTs.

### 2.4. Three-Point and Four-Point Bending Test

Three-point (3-P) bending test specimens of 40 mm × 40 mm × 160 mm after curing seven days and 28 days with a 20-mm initial cut were deposited onto an AG-TS testing machine (Shimadzu, Kyoto, Japan). The initial crack–height ratio was 0.5 and the bearing spacing was 150 mm. Two LDVTs affixed with a rigid support installed on the lateral faces of the specimen were used to determine the deflection, and load change was acquired by the data acquisition system provided by the testing machine.

For the four-point (4-P) bending test, a similar sequence was followed; the only difference was the bending configuration, which allowed part of the specimen between the two loads to be subjected to a pure bending constant effort. The spacings between the two loads and the two supports were 50 mm and 150 mm, respectively.

### 2.5. Capillary Absorption Test

Cement mortar cubes (70.7 mm × 70.7 mm × 70.7 mm), which were cured for 28 days in a standard curing room, were dried in an oven at 60 °C until constant weight was reached. The four side surfaces of the dry specimens, sealed with aluminum foil ensuring one-dimensional water absorption, were then brought into contact with water. The specimen bottom was kept constant at approximately 3 mm below the water surface using a special support. The mass of the specimens was weighed at 0.5 h, 1 h, 2 h, 4 h, 8 h, 12 h, two days, three days, seven days, and 28 days.

### 2.6. Chloride Penetration Test

A similar sequence was followed by the chloride penetration test, except that the water in the container was replaced with an aqueous 3.5% NaCl solution. After 60 days, specimens were milled consecutively to a depth of 30 mm with thin layers of 1 mm thickness from the surface to 10 mm and 2 mm thickness from 10 mm to 30 mm, respectively. The chloride content of the powder was finally determined by titration.

## 3. Results and Discussion

### 3.1. Effect of Aqueous Alkali on Natural Plant Fibers

The main drawback associated with natural plant fibers in cement applications is their durability in cementitious composites [11]. The cement matrix is a strongly alkaline material caused by the hydration process of cement, which produces a considerable amount of calcium hydroxide [39]. In this section, the effects of aqueous alkali on the surface morphology and tensile strength of natural plant fibers were analyzed.

**Figure 5:**
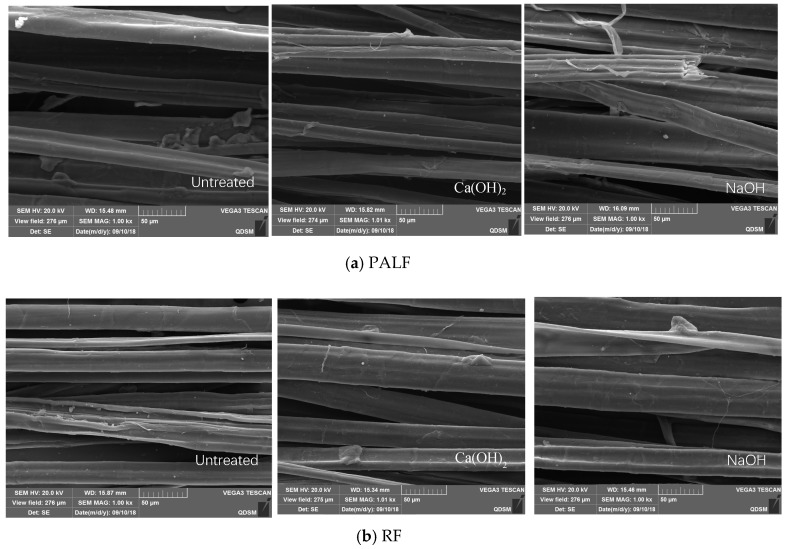
SEM images of PALF and RF before and after being soaked in Ca(OH)_2_ and NaOH solutions with a pH value of 12.

The surface morphology of natural plant fibers was obtained using a TESCAN VEGA3 scanning electron microscope, as shown in Figure 5. The untreated fibers and fibers treated with aqueous alkali for 60 days were dried in an oven at 100 °C and sprayed gold before the experiment.

It can be seen from Figure 5 that the monofilament fibers of both types were not uniform, with branches in the lateral direction and cavities in the middle. Both fibers had a smooth surface following the degumming process which removed surface impurities. The smooth surface was proven to be effective in reducing the chemical and frictional bonding between the fiber and cementitious matrix, and, in this case, the specimens revealed pull-out behavior instead of fiber rupture, which was more favorable in terms of energy adsorption [40]. The cementitious matrix incorporated with natural plant fibers with a smooth surface was expected to adsorb more energy. The discussion presented in Section 3.4 may support this point. Other than fiber length and aspect ratio, comprehensive studies showed that the bond behavior between the fiber and cement matrix was of significant importance for the final properties of the composites, particularly flexural toughness [29] It is of interest to note that the surface morphology did not change significantly before and after being soaked in the two aqueous alkaline solutions.

**Figure 6:**
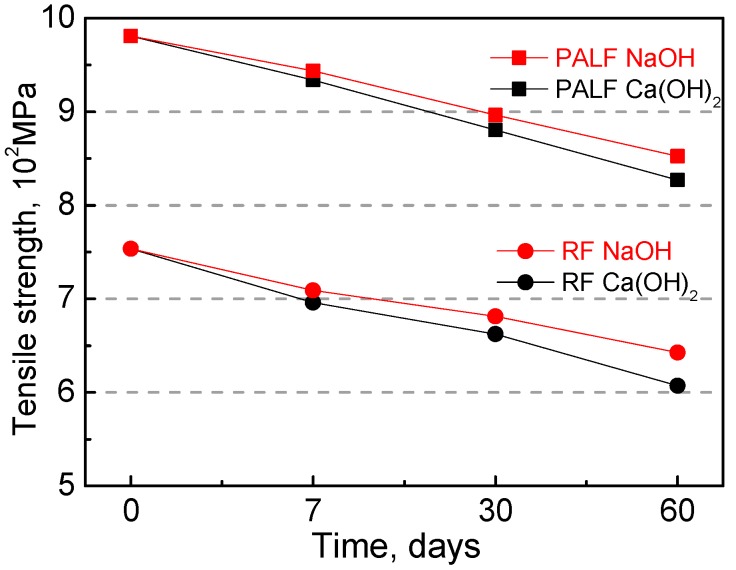
Tensile strength of natural plant fibers.

The tensile strength of natural plant fibers was measured after firstly soaking in Ca(OH)_2_ and NaOH solutions for seven days, 30 days, and 60 days, and then drying in an oven at 100 °C for one day. Typical results are shown in Figure 6. All mix ratios showed reduced tensile strength as the soaking age increased, with the worst 60-day performance coming from RF soaked in the Ca(OH)_2_ solution. It is of interest to note that PALF outperformed RF in terms of alkali resistance, and the damage degree was smaller when fibers were immersed in the NaOH solution.

The main components of natural plant fibers are cellulose, hemicellulose, and lignin, whereby cellulose being prone to hydrolysis in highly alkaline solutions plays a key role in fiber performance [12]. The reduction in tensile strength of natural plant fibers can be explained by the degradation of cellulose molecular chains, which induces a decline in polymerization degree.

### 3.2. Mechanical Properties

**Figure 7:**
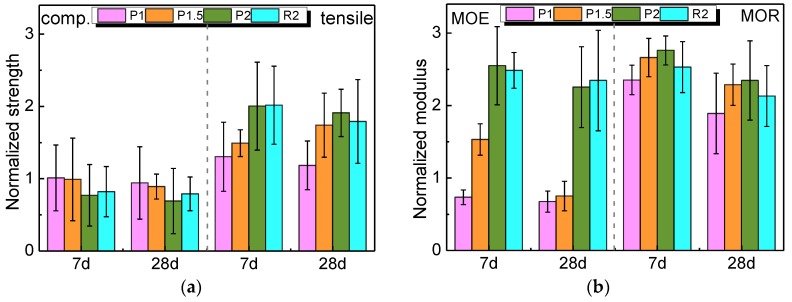
Normalized compressive strength, tensile strength, modulus of elasticity (MOE), and modulus of rupture (MOR) of NPFRCCs at seven days and 28 days.

Figure 7 outlines the proportional performance of the NPFRCCs relative to the plain control (C0). For each of the properties, the normalized values at seven days and 28 days are given as the ratio of the NPFRCC performance to the performance of C0.

Figure 7 shows that all fiber ratios at seven days exhibited a reduction in compressive strength compared to the control, and a significant reduction can be seen for P2. A similar decreasing tendency was also found in all specimens at 28 days, where P2 exhibited a 31% reduction. The compressive strength decreased with increased PALF content, and RF produced a higher compressive strength than PALF. The voids and air pockets introduced by natural plant fibers was likely responsible for the observed strength reduction. Fiber-reinforced composites had more pores and a larger pore size compared to the plain control [41]. It was also observed that the interfacial transition zone (ITZ) between the matrix and fiber comprises a weak layer [39]. The porous ITZ of NPFRCCs could also contribute to the strength loss.

The tensile strength showed an entirely different changing rule compared to the compressive strength. All specimens showed a prominent strength enhancement with the increase in fiber content, and the maximal enhancement was found in P2 with a 200% increase at seven days. A similar strength increase was presented in PALF and RF. The direct tensile strength depends largely on the material’s post-peak behavior [42]. Since fibers are primarily engaged after the occurrence of cracking, the tensile strength of the NPFRCC was expected to significantly increase.

The MOE of PALF-reinforced composites exhibited an increasing tendency with increased fiber incorporation, although P1 at seven days and P1 and P1.5 at 28 days presented values below the MOE of C0.

As with the tensile strength and MOE, MOR was also observed to enhance with the increase in PALF incorporation. Unlike the aforementioned parameters, higher enhancement was gained in MOR, with an approximately 250% increase at seven days and a 200% increase at 28 days. RF-reinforced composites exhibited a slight reduction in MOR compared to the PALF-reinforced composites.

It should be noted that RF produced a higher compressive strength and a slightly lower tensile strength as compared to PALF. The same changing rule was also found for MOE and MOR. The larger ultimate elongation and the larger aspect ratio of PALF were likely responsible for this phenomenon [29].

### 3.3. Tensile Stress–Strain Behavior

**Figure 8:**
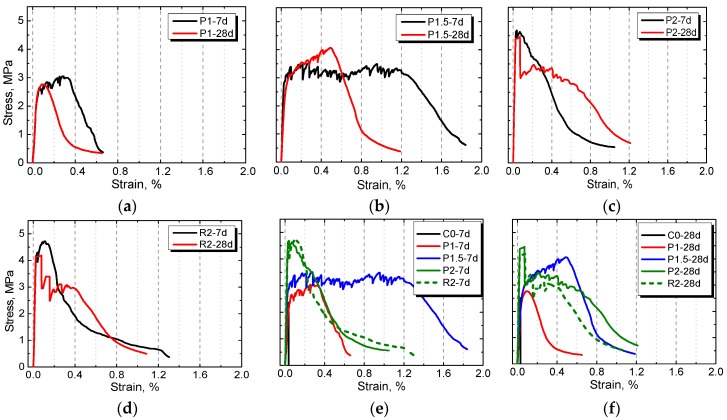
Tensile stress–strain behavior of NPFRCCs with different amount of fibers ((**a**) P1, (**b**) P1.5, (**c**) P2 and (**d**) R2) and the comparison at (**e**) 7 days and (**f**) 28 days.

Figure 8 illustrates the tensile stress–strain behavior of the plain control and NPFRCCs at seven days and 28 days. Obviously, the material’s failure mode went from brittle toward the more favorable ductile due to the incorporation of PALF. Among all the ratios, the strain-hardening phenomenon was more predominant with higher tensile strength and higher tensile capacity with a PALF content of 1.5% at seven days. As with a previous study [43], the failure process could be divided into three distinct branches: A linearly ascending branch, a nearly horizontal branch, and a descending branch. In the first branch, the external load was mainly undertaken by the cement matrix, and the interior micro-cracks were slowly developed. In the horizontal branch, when the matrix was cracked, the load then transferred to the fibers, which continued resisting load as long as they were not ruptured or pulled out. Meanwhile, the load was transferred to the matrix nearby the crack, hindering the crack’s further development. Owing to the load cycle, fluctuation was observed in the stress–strain curve, where each wave represents a new crack. During the descending phase, the specimen exhibited a strain-softening characteristic with further expansion of cracks. The width of the micro-crack at the weakest point increased, eventually forming a main crack, which caused the specimen to break.

It should not be ignored that a more ductile performance was observed in NPFRCCs at seven days rather than 28 days, illustrating that NPFRCCs had a better performance at an early curing age. PALF-reinforced composites had a relatively better performance compared to RF-reinforced composites; however, a wider range of fiber content still needs to be evaluated, since only a relatively high fiber incorporation was tested in this paper.

### 3.4. Load–Deflection Curves

**Figure 9:**
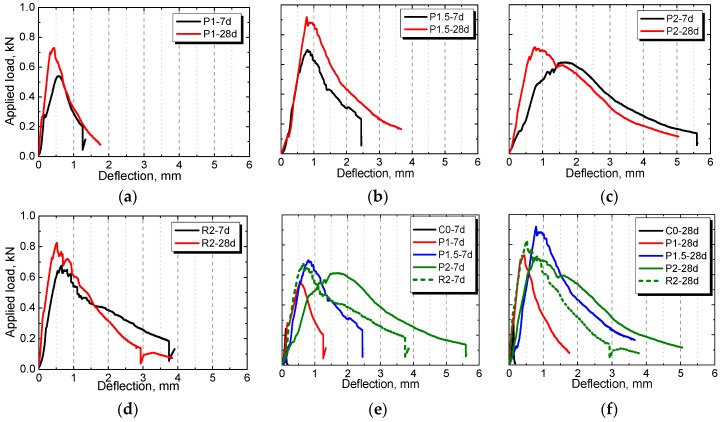
The load–deflection curves of NPFRCCs with different amount of fibers ((**a**) P1, (**b**) P1.5, (**c**) P2 and (**d**) R2) and the comparison at (**e**) seven days and (**f**) 28 days based on the three-point (3-P bending test.

The load–deflection curves of the plain control and NPFRCCs at seven days and 28 days based on the 3-P bending test were obtained under the influence of fiber content and fiber type. Typical results are shown in Figure 9. It can be seen that, firstly, deflection rose rapidly with the increase in load; then, fine cracks initiated on top of the reserved crack when the load reached its peak. Afterward, the deflection continued to increase in response to the expansion of fine cracks, which facilitated the load’s slow decline. Finally, the specimen fractured when the crack developed to a certain degree. The incorporated fiber caused the peak load to drop at a very slow rate compared with the plain control, indicating that fiber-incorporated specimens consumed a large amount of energy during the destruction process. Only when the load dropped to a considerably lower value did the specimen show abrupt brittle fracture, implying that natural plant fibers in the cement matrix were insufficient to withstand the tensile force between the cracks, and they pulled out or ruptured as a result.

**Figure 10:**
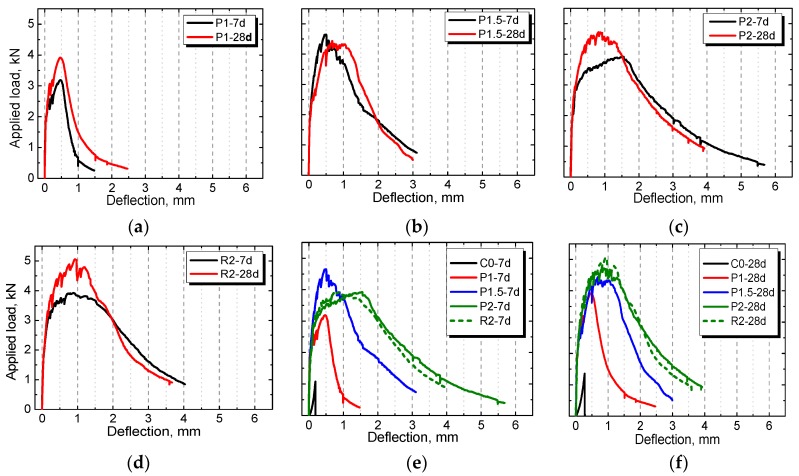
The load–deflection curves of NPFRCCs with different amount of fibers ((**a**) P1, (**b**) P1.5, (**c**) P2 and (**d**) R2) and the comparison at (**e**) 7 days and (**f**) 28 days based on the 4-P bending test.

The load–deflection curves of the plain control and NPFRCCs at seven days and 28 days based on the 4-P bending test were obtained under the influence of fiber content and fiber type. Results are shown in Figure 10. It is known that fibers do not necessarily improve the pre-crack behavior at which cracking occurs; however, the improvement is evident in the post-crack behavior of the composites [42]. A similar pre-crack behavior of NPFRCCs was expected since all specimens contained similar matrices. On the other hand, the post-crack response differed due to the different fiber types and content. The plain control continuously broke; however, the fiber-reinforced composites exhibited superior deformation ability.

Apparently, the 4-P bending test allows bearing more loads, which is attributed to its configuration that allows samples between the two loads to be subjected to a pure bending constant effort, thereby avoiding the break point, ensuring that the correct results are obtained. The 3-P bending test can still be used to determine the MOR and fracture energy, though the load squashes the surface of the specimen.

### 3.5. Fracture Energy

**Figure 11:**
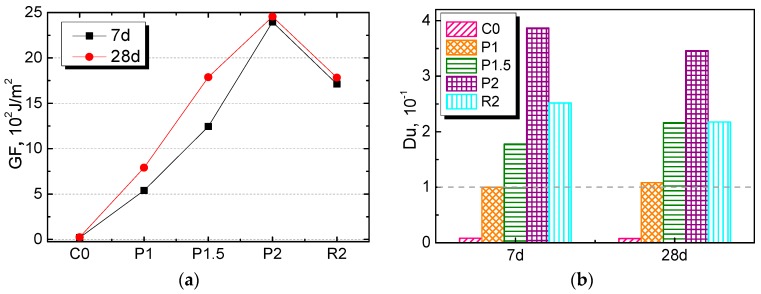
Fractural properties: (a) fracture energy and (b) ductility index of NPFRCCs at seven days and 28 days.

Figure 11 shows the influence of fiber type and content on the fracture energy (GF) and ductility index (Du) at seven days and 28 days. It can be seen that GF and Du continued increasing with the increased PALF content, whether at seven days or 28 days, indicating that PALF improved the ability to resist fracture; this enhancement became more significant as the fiber content increased. Comparing the PALF- and RF-reinforced composites with the same content of 2%, it can be found that PALF-reinforced specimens exhibited a higher fracture energy and ductility index, illustrating that PALF had a better reinforcing effect on cement-based materials. In addition, curing age was also an important factor affecting the fractural properties of the composite material. The fracture energy of each specimen at 28 days was higher than that at seven days, which was largely due to the subsequent cement hydration. However, the ductility index declined slightly as curing age increased.

### 3.6. Flexural Toughness

The flexural toughness evaluation index is the mostly widely used indicator for measuring the toughness of fiber-reinforced cementitious composites. Many countries developed test method standards, such as ASTM C1018, ASTM C1609, JSCE-SF4, and RILEM TC 162-TDF, which define the flexural toughness evaluation index of fiber-reinforced composites from various perspectives [44]. The RILEM TC162-TDF standards are not used here owing to the great difference between the test methods and loading system compared to those used in this paper. Attention was paid to the other methods, and the flexural toughness parameters are shown in Table 4.

**Figure 12:**
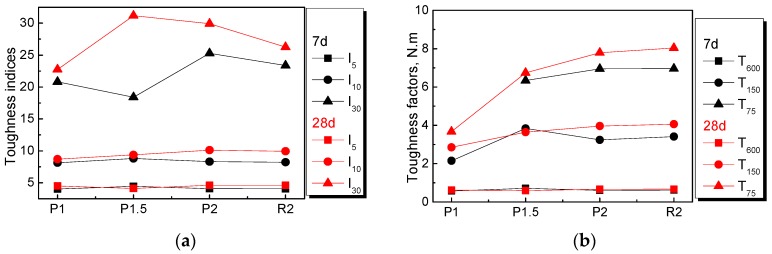
Flexural toughness parameters: (**a**) toughness indices and (**b**) toughness factors of NPFRCCs at seven days and 28 days.

The flexural toughness parameters of NPFRCCs at seven days and 28 days were determined using the ASTM C1608 method, ASTM C1609 method, and JSCE-SF4 method; the results of the former two are shown in Figure 12, and those of the latter are shown in Table 4.

From the results shown in Figure 12 and Table 4, it can be observed that flexural toughness indices I_5_, I_10_, and I_30_ varied considerably based on the ASTM C1018 method, while the toughness factors calculated using the ASTM C1609 method presented a growing tendency with increased PALF content, which is in agreement with the results shown in Figure 10. The same conclusion was drawn using the JSCE-SF4 method. For the ASTM C1609 method, in the case of a small deflection, seen as T_600_, the differences in the toughness factors of all mix ratios were not significant, while, in the case of moderate and large deflections, the toughness factors improved remarkably with increased fiber content. The improvement in flexural toughness was attributed to the smooth fiber surface, which allowed fiber pullout instead of rupture. It should also be noted that the toughening effect was better at 28 days than at seven days, which was attributed to the subsequent cement hydration process. In summary, the ASTM C1608 method, closely associated with the initial crack, where a small error would have a great influence on the final result, is inadaptable to evaluate the flexural toughness of NPFRCCs. In contrast, it is clear that yje ASTM C1609 and JSCE-SF4 methods are more suitable for evaluating the flexural toughness of NPFRCCs.

### 3.7. Capillary Absorption

**Figure 13:**
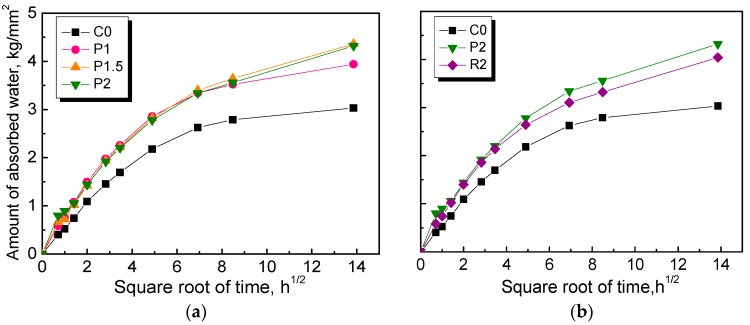
Influence of fiber content (**a**) and fiber type (**b**) on the water capillary absorption of NPFRCCs.

The amount of capillary water absorption was measured, and the test results are shown in Figure 13.

If gravity is neglected, the amount of capillary-absorbed water as a function of time can be described using the following equation [45,46]:

(1) $$\Delta W\left(t\right)=A\sqrt{t}$$

The water absorption process can be described in a simplified way using Equation (2), and the parameters a and b can be determined by fitting Equation (3) with experimental data, as shown in Figure 13 [47,48].

(2) $$\Delta W\left(t\right)=a\left[1-e^{-b\sqrt{t}}\right]$$

(3) $$A\left(t\right)=\frac{d\Delta W}{dt}=\mathrm{ab}e^{-b\sqrt{t}}$$

**Figure 14:**
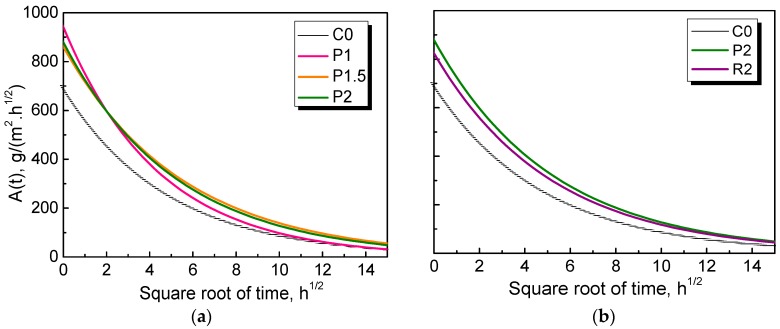
Time-dependent coefficient of capillary absorption of (**a**) PALF reinforced composites and (**b**) comparison of C0, P2 and R2.

The time-dependent coefficient of capillary absorption *A*(t) of NPFRCCs was then calculated. The results are shown in Figure 14. It is obvious that the coefficients for all mix ratios increased when the samples came into contact with water, and a constant lower value was eventually reached. It is also of interest to note that the coefficients of NPFRCCs were significantly larger than that of the plain control, with the worst performance coming from P2, which was attributed to the increased channels provided by the randomly dispersed fibers that aggravated the intrusion of water. Moreover, PALF-reinforced cementitious composites exhibited a relatively higher water absorption value than the RF-reinforced composites.

### 3.8. Chloride Penetration

**Figure 15:**
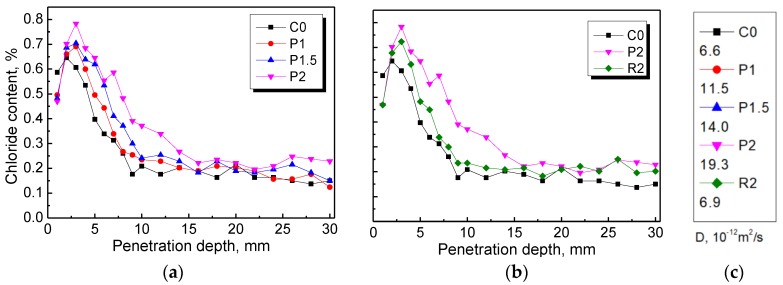
The influence of fiber content (**a**) and fiber type (**b**) on the resistance to chloride erosion of NPFRCCs. The coefficient of chloride diffusion (D) (**c**) of NPFRCCs.

The relationship between chloride content and penetration depth of the plain control and NPFRCCs is shown in Figure 15a,b, and the coefficients of chloride diffusion are shown in Figure 15c. It was mentioned in Section 3.7 that the amount of water, absorbed by capillary action per unit time, increases due to the increased channels provided by the randomly dispersed fibers. As a result, NPFRCCs exhibited higher chloride content owing to the more dissolved chloride. It is worth noting that the highest chloride concentration was reached near the surface (2 mm and 3 mm) of the specimens for the plain and natural plant fiber-reinforced specimens, respectively. The aforementioned phenomenon may be a result of the nanopores acting as a molecular filter, thereby hindering the penetration of the dissolved chloride into the pore space [47,49,50]. Then, a slow diffusion process would ensure, due to the enormous concentration difference in the number of chloride ions between the near-surface and the deeper zones. As a consequence, the penetration depths of the plain control and the natural plant fiber-reinforced composites were approximately 10 mm and 16 mm, respectively. In addition, the coefficient of chloride diffusion of NPFRCCs increased with increased PALF content, in accordance with the results shown in Figure 15a. The coefficient of chloride diffusion of P2 being approximately three times bigger than that of R2 indicates that RF outperformed PALF in terms of resistance to chloride ion erosion, although it was inferior to the plain control.

## 4. Conclusions

The effects of PALF and RF on the mechanical properties and mass transport of cement-based composites were studied. Based on the results presented herein, the below conclusions can be drawn. 

NPFRCCs containing PALF and RF exhibited a slight reduction in compressive strength compared to the plain control. A marginally larger reduction was observed in the cement composites with a higher fiber content. In contrast, a considerable improvement was found in the tensile strength, MOE, and MOR. The specimen with 2% PALF content exhibited optimal mechanical properties. 

Despite the reduction in compressive strength, there was a notable improvement in flexural properties. Natural plant fibers substantially improved the flexural toughness of cement-based composites with an optimum PALF content of 2%. Meanwhile, natural plant fibers were also beneficial to the improvement in fracture energy of cementitious composites with an optimum PALF content of 1.5%.

The coefficients of capillary absorption and chloride diffusion of NPFRCCs were significantly larger than those of the plain control, and the differences were evident with the increase in PALF fiber content.

In summary, the present study suggests that the specimen with 2% PALF content can be used in normal environments due to its superior mechanical properties. However, one should be careful when using the material in marine environments. To better evaluate its feasibility, detailed research studies with respect to improving the performance of PALF- and RF-reinforced cement-based composites are required to expedite industry uptake.

## Figures and Tables

**Table 1:** Compositions of all five types of mortar used in this project.

Type	Cement (kg/m³)	Fly Ash (kg/m³)	Sand (kg/m³)	Water (kg/m³)	PALF (vol.%)	RF (vol.%)	PC (wt. %)
C0	550	650	550	395	0	-	0.22
P1	550	650	550	395	1	-	0.33
P1.5	550	650	550	395	1.5	-	0.55
P2	550	650	550	395	2	-	0.87
R2	550	650	550	395	-	2	0.65

Note: PC, polycarboxylic acid superplasticizer; P, pineapple leaf fiber (PALF); R, ramie fiber (RF); wt.% is the weight fraction of cement.

**Table 2:** Chemical composition of ordinary Portland cement (OPC) and fly ash (FA) (%).

Type	SiO_2_	Al_2_O_3_	Fe_2_O_3_	CaO	MgO	SO_3_	K_2_O	Na_2_O	TiO_2_	P_2_O_5_
OPC	22.91	7.35	3.1	57.46	4.07	1.52	0.47	0.99	0.35	0.05
FA	52	26.68	4.5	8.07	1.18	1.14	1.54	-	-	-

Note: OPC: Ordinary Portland cement; FA: Fly ash.

**Table 3:** Properties of fibers.

Type	Diameter (μm)	Length (mm)	Elastic Modulus (GPa)	Fiber Strength (MPa)	Ultimate Elongation (%)	Density (g/cm^3^)
PALF	20–50	30	10.78	980.9	3.42	1.54
RF	25–40	30	9.99	753.4	3.23	1.25

Note: The tested fiber length was 30 mm, while the fiber used in natural plant fiber-reinforced cement-based composites (NPFRCCs) was 15 mm due to the limitation of the automated monofilament fiber tester, which can only test the standard lengths of 10, 20, and 30 mm.

**Table 4:** Flexural toughness parameters in four-point (4-P) bending test.

Standards	Parameters	P1	P1.5	P2	R2
ASTM C1018	I_5_	4.02 (4.50)	4.45 (4.11)	4.07 (4.60)	4.05 (4.61)
	I_10_	8.13 (8.69)	8.80 (9.38)	8.31 (10.11)	8.22 (9.94)
	I_30_	20.82 (22.74)	18.41 (31.17)	25.27 (29.91)	23.36 (26.25)
ASTM C1609	T_600_ (N∙m)	0.57 (0.62)	0.71 (0.59)	0.61 (0.66)	0.62 (0.67)
	T_150_ (N∙m)	2.15 (2.85)	3.84 (3.64)	3.24 (3.96)	3.41 (4.06)
	T_75_ (N∙m)	−(3.67)	6.34 (6.74)	6.95 (7.79)	6.96 (8.04)
JSCE-SF4	*f*_e_ (MPa)	5.03 (6.67)	8.99 (8.52)	7.58 (9.27)	7.98 (9.50)

Note: The 28-day and seven-day parameters are indicated inside and outside the brackets, respectively. T_600_ is defined as the area under the load–deflection curve up until a midpoint deflection equal to 1/600th of the flexural span.
